# Astrocyte to neuron reprogramming with NeuroD1 for repair in canine stroke

**DOI:** 10.3389/fstro.2025.1602076

**Published:** 2025-07-02

**Authors:** Isaac H. Clark, Zachary Roushdy, Dilmareth Natera-Rodriguez, Kevin Sun, Olivia Erlanson, Shaimaa Khedr, Walter C. Low, Andrew W. Grande

**Affiliations:** ^1^Biomedical Engineering Graduate Program, University of Minnesota, Minneapolis, MN, United States; ^2^Department of Neurosurgery, University of Minnesota, Minneapolis, MN, United States; ^3^Stem Cell Institute, University of Minnesota, Minneapolis, MN, United States; ^4^Graduate Program in Neuroscience, University of Minnesota, Minneapolis, MN, United States

**Keywords:** stroke, NeuroD1, astrocyte, canine, adeno-associated vector (AAV)

## Abstract

Stroke affects hundreds of thousands of people each year and leads to neuronal damage and often long term disabilities. This study served as an exploratory study investigating astrocyte to neuron reprogramming as a potential treatment for ischemic stroke using a canine model to assess anatomical and functional recovery. The study's exploratory nature involved a small sample size, precluding statistically significant conclusions. For treatment, an adeno associated viral (AAV) vector was constructed such that it would target astrocytes and allow expression of NeuroD1 for the intent of reprogramming them into neurons. Animals were analyzed anatomically using MRI scanning, behaviorally with neurological severity score testing, and cellularly with immunohistochemistry staining. Behaviorally, treated animals recovered more rapidly and to a greater extent than controls; anatomically, treated animals also showed much less ventricle enlargement post stroke; and on the cellular level, treated animals showed a decreased level of astrocyte and microglial activation. These findings suggest that NeuroD1-mediated astrocyte reprogramming may reduce neuroinflammation and enhance functional recovery in ischemic stroke, warranting further exploration of this therapeutic approach.

## 1 Introduction

Stroke afflicts more than 700,000 people each year in the US alone (Tsao et al., 2023). Of those who survive stroke, nearly two thirds of them develop a long term disability. Stroke occurs when the supply of blood to the brain tissue is disrupted due to a focal vascular cause, resulting in neuronal damage (Knight-Greenfield et al., 2019). Stroke can be divided into two major categories, ischemic, which occurs when a blockage within the blood vessels prevents blood flow to a portion of the brain, and hemorrhagic stroke, which occurs when a blood vessel ruptures and leaks blood into a portion of the brain. The ischemic form of stroke can be modeled in animals through clamping a major vessel, such as the middle cerebral artery, and preventing blood flow for a period of time. Due to their size, brain structure, and vascular system, canine models can provide an effective representation of the pathophysiological effects of transient occlusions and subsequent molecular events that can be translated to the human condition (Lv et al., 2020). Canines have an established circle of Willis, cortical folding, and additional complexities similar to humans. This makes them a more accurate model compared to rodents that do not share these qualities (Schröder et al., 2020). While more similar models to humans exist, their use often raises additional ethical concerns, and regulatory challenges. Canines provide an ideal model that is often more accessible to researchers.

Neurons have a very limited ability to recover post injury due to an inability to divide and regenerate. Processes such as neurogenesis can promote recovery, but little is understood about this process and it is typically limited to the hippocampus and rostral migratory stream (van Strien et al., 2011; Cameron and Glover, 2015). Cell reprogramming, such as astrocyte to neuron reprogramming, is a process that has the potential to treat neurological diseases and disorders by replacing damaged or diseased neurons with new neurons originating from astrocytes (Peng et al., 2022). This process is often performed through the use of an Adeno Associated Viral (AAV) vector that targets astrocytes by using promoters that are highly active in astrocytes such as the glial fibrillary acidic protein (GFAP) promoter. GFAP is expressed in both resting (inactive) and reactive (active) astrocytes. However, its expression is significantly upregulated in reactive astrocytes, particularly in response to brain injuries such as stroke (Eng and Ghirnikar, 1994). Stroke and other forms of neural injury thus tend to increase the number of reactive astrocytes in the affected area. While reactive astrocytes can support tissue repair, they may also produce excessive levels of cytokines, which can become neurotoxic. Targeting astrocytes near the injury site for conversion into functional neurons, allows for the potential replacement of damaged neurons, enhanced neurological recovery, and reduction of the production of harmful cytokines.

Cellular reprogramming of astrocytes into neurons has been studied in stroke models such as rats with anatomical and neurological benefits being recorded (Chen et al., 2020). This process has also, however, been riddled with controversy as to the origin of the resulting neurons. Lineage tracing, viral concentration (dose), viral serotype, and alternate promoters all have the potential to affect the apparent transduction and cellular changes. AAV transduction at higher doses has been shown to result in transaminase elevations and cell degeneration (Hinderer et al., 2018). If astrocytes are targeted for transduction, this can result in the death of said astrocytes, leakage of the promoted biomarkers, and confounding results regarding the cells that express the promoted biomarkers (Xu et al., 2022). To explore cellular reprogramming in a more translatable stroke model of cerebral ischemia, we induced cellular reprogramming in canine stroke models. The study's exploratory nature involved a small sample size, precluding statistically significant conclusions. In this exploratory study, we found promising results of anatomical and behavioral recovery that should encourage further research in the field.

## 2 Methods

### 2.1 Animals

The three animals used in this study were male canine beagles with weight 8 to 11 kg. The sample size for this study was intentionally kept small to align with its exploratory nature. The insights gained from this pilot investigation are intended to guide the design of a future study, informed by the variance and effect sizes observed here. All animals underwent an induced stroke procedure. Due to inherent procedural variability, one of the animals developed a significantly smaller infarct area compared to the others. Based on this outcome, the animals were allocated into two groups:

- Treatment Group (*N* = 2): One canine with a large stroke and one with a small stroke. Both injected with AAV9-GFAP(short)-Cre + AAV9-CAG-DIO-NeuroD1-T2A-mRuby2.
- Control Group (*N* = 1): One canine with a large stroke. Injected with AAV9-GFAP(short)-Cre + AAV9-CAG-DIO-mRuby2.

No negative controls (canines without treatment or control virus injection) were used in this study, as one of the primary objectives was to assess the transduction properties of the viral vector both with and without neuronal reprogramming. We anticipated that the control virus would primarily label astrocytes with mRuby2, while the treatment virus would preferentially label neurons, indicating successful neuronal reprogramming. Our viral system was adapted from Chen et al., who previously demonstrated this transduction specificity (Chen et al., 2020).

### 2.2 Virus

**Figure 1:**
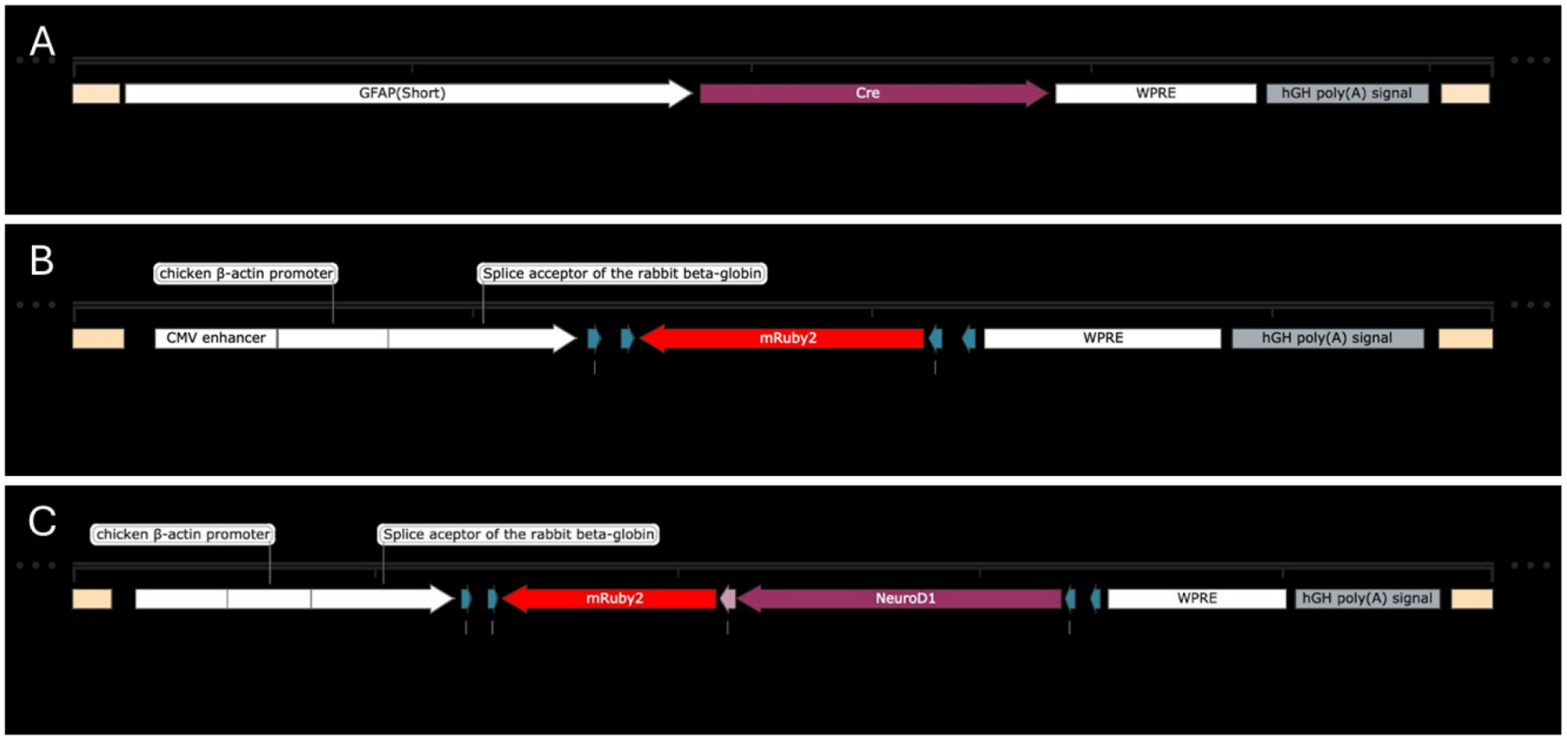
Transgenes for astrocyte transduction and reprogramming. **(A)** AAV9-GFAP(short)-Cre. **(B)** AAV9-CAG-DIO-mRuby2. **(C)** AAV9-CAG-DIO-NeuroD1-T2A-mRuby2.

To induce reprogramming, we used a set of serotype 9 Adeno Associated Viral (AAV9) vectors (which have been shown effective in transducing neuronal tissues). The first vector, which was used in both treated and control animals, was AAV9-GFAP(short)-Cre which was given at a dose of 2.4E13 GC/mL (Figure 1A). The vector was designed to induce expression of Cre recombinase specifically in astrocytes via a GFAP promoter. Cre recombinase alters DNA with specific sequences known as loxP sites and can invert sequences that are flanked by the sites, viable for protein synthesis. In our case, this was used in combination with a second control or treatment vector containing a transgene with loxP sites, which would allow for the transgene to be expressed. All viral systems were administered 7 days after stroke induction. This timing was selected to coincide with the peak of astrocyte proliferation and activation following stroke, maximizing the population of astrocytes available for targeting (Barreto et al., 2011). This approach is intended to enhance transduction efficiency and optimize the therapeutic potential of the vector.

The control secondary vector was AAV9-CAG-DIO-mRuby2, which was given at a concentration of 2.5E13 GC/mL (Figure 1B). The CAG promoter was meant to induce expression of the desired genes in any cells that were also transduced by the first virus. This promoter is independent of cell type, thus allowing the following sequence to continue to be produced even after reprogramming may change the cell phenotype from an astrocyte to a neuron. The mRuby2 biomarker was meant to label all transduced cells. The secondary treatment vector was AAV9-CAG-DIO-NeuroD1-T2A-mRuby2, which was delivered at a concentration of 1.0E13 GC/mL (Figure 1C). This viral system was meant to function in the same manner as the control with the addition of allowing expression of NeuroD1 in transduced cells. NeuroD1 is a transcription factor that has been shown in numerous studies to induce cellular reprogramming, and in this study, it was meant to convert astrocytes into neurons.

### 2.3 Canine stroke

The canine stroke model we used was established previously within our lab (Vasquez et al., 2019). On the day of the procedure, each animal is sedated with Acepromazine, 0.05–0.2 mg/kg, IM. Two 18-guage IV's are placed in either fore or hind limbs depending on vein accessibility. Propofol, 4–6 mg/kg, is administered IV. Canines are then intubated and maintained under general anesthesia using isoflurane (1.5%−2.5%) for the duration of the procedure. During this time vital signs including pulse, blood pressure, and temperature are monitored according to IACUC specifications and APIC standards. Additionally, prior to beginning the procedure, eye lubricating ointment is applied to both eyes, the eyes are closed, and the animals receive a 30 mg/kg dose of Cefazolin. After each animal was anesthetized, the right frontal region of the head was shaved, and prepped with alcohol then betadine. Sterile towels were then draped around the surgical site in the right frontal area.

A curvilinear incision, measuring between 1–2.5 inches in length, was made approximately 1 cm above the level of the right zygomatic arch. The skin was then reflected inferiorly and held in place by a suture. Hemostasis was obtained with bipolar and monopolar cautery. The zygomatic arch was identified and removed using a pneumatic drill. Bleeding from the bone was controlled with bone wax. The temporalis muscle was incised using a monopolar cautery in a linear fashion, perpendicular to the previous zygomatic arch, exposing the underlying cranium. After mobilizing the temporalis muscle off the cranium with a periosteal dissector, the muscle was held in place with fishhook retractors. Using a pneumatic drill, a 2 cm × 2 cm area of bone was removed at the inferior level of the temporal lobe. Care was taken to drill the cranium down to the level of the middle fossa floor so that retraction of the temporal lobe can be minimized. The underlying dura was then coagulated with bipolar cautery and opened with either a scalpel or an insulin syringe.

A neurosurgical microscope, sterilely draped, was then brought into the surgical field for the remainder of the procedure. Using microscopic dissection techniques, the frontal lobe was gently retracted and held in place with a small brain retractor. The arachnoid over the frontal lobe was incised with an insulin syringe. The distal middle cerebral artery (MCA) was identified and traced back to the proximal or M1 segment where it divides from the internal carotid artery (ICA). Next the ophthalmic artery (OA) and anterior cerebral artery (ACA) were isolated. At this time aneurysm clips were temporarily applied to the ACA, OA and the MCA (distal to the lenticulostriate vessels). These clips remain in place for 1 h. After placing the aneurysm clips the MCA pial collateral vessels are identified and coagulated. This step was essential to minimize collateral circulation to the intended area of infarction. Once 60 min passed, the 3 aneurysm clips were removed. After irrigating the surgical site, the temporalis muscle was closed with interrupted or running sutures followed by a primary skin closure using either interrupted or running sutures.

### 2.4 Virus injection

Animals were prepped and anesthetized in the same manner as the stroke procedure. Following exposure of the previous surgical area, the virus was prepped for injection. Each injection consisted of 0.5 μL of AAV9-GFAP(short)-Cre with either 0.5 uL of AAV9-CAG-DIO-mRuby2 or 0.5 uL of AAV9-CAG-DIO-NeuroD1-T2A-mRuby2. Five injections were given surrounding the peripherals of the stroke areas at a depth of ~1 cm. After the injection, the site was closed in the same manner as the stroke procedure.

### 2.5 Behavior

To assess neurological deficits and functional recovery, a modified neurological severity score was acquired based on several neurological assessments as seen in Table 1. All tasks were assessed by visual observation unless otherwise stated. Scoring systems such as these have been used by other research groups in canine models and have been proven successful (Snyder et al., 2015; Rosenthal et al., 1992). Scoring categorized as severe, moderate, or modest, was determined by an experienced researcher using standardized criteria and/or their professional judgment.

**Table 1:** All behavioral tests used to generate the Canine neurological severity score.

**Test**	**Scores**
Stance	(1 = broad based stance, 0 = normal stance)
Cerebellar ataxia	(3 = severe gait dysmetria, 2 = moderate gait dysmetria, 1 = modest gait dysmetria, 0 = normal gait);
Unilateral vestibular ataxia: leaning or falling to one side	(3 = severe, 2 = moderate, 1 = modest, 0 = no leaning and falling)
Unilateral vestibular ataxia: head tilt	(3 = severe, 2 = moderate, 1 = modest, 0 = no head tilt)
Unilateral vestibular ataxia: nystagmus	(3 = severe, 2 = moderate, 1 = modest, 0 = no nystagmus)
Bilateral vestibular ataxia	(3 = severe, 2 = moderate, 1 = modest, 0 = none)
Hemiparesis	(3 = severe, 2 = moderate, 1 = modest, 0 = none)
Paraparesis	(3 = severe, 2 = moderate, 1 = modest, 0 = none)
Proprioceptive positioning: (assessed by inverting paw, placing dorsal surface in contact with ground and timing till corrected) (left/right, fore/hind)	(2 = severe delay, 1 = moderate delay, 0 = normal)
Placing response: (assessed by covering vision, moving limb to edge of surface, and timing till reflexively placed upright on surface) (left/right, fore)	(2 = severe delay, 1 = moderate delay, 0 = normal)
Placing response visual	(assessed by moving limb to edge of surface and timing till reflexively placed upright on surface) (left/right, fore) (2 = severe delay, 1 = moderate delay, 0 = normal)
Tremor	(3 = severe, 2 = moderate, 1 = modest, 0 = none)
Auditory response	(Assessed by clapping on left or right side and recording response) (Ipsilateral/Contralateral) (1 = no response to noise, 0 = normal orientation to noise)
Best ambulation attempt	(8 = no movement, 7 = unable to right self but moves, 6 = able to right self, 5 = unable to stand, 4 = stands w/assistance, 3 = stands w/o assistance, 2 = circles but falls to side, 1 = circles, 0 = normal)
Vocalization	(2 = none, 1 = howls/grunts, 0 = normal)
Consciousness	(3 = does not awaken, 2 = awakens w/noxious stimulus, 1 = awakens w/minimal stimulation, 0 = awake & interactive)

### 2.6 MRI acquisition

MRIs were obtained in the University of Minnesota MRI (located in the basement of the hospital which is connected to PWB RAR facility by tunnel). Animals were anesthetized with general anesthesia for MRI. The scan tools are approximately 1 h and include T1, T2, T2^*^ gradient echo, flair, TOF, and perfusion scans. Once stable, animals were transported from MRI to a holding cage and recovered by RAR care staff.

### 2.7 Anatomical quantification

To quantify stroke lesion volume, the T2 scans were loaded into 3D slicer and segmented. Segmentation was performed using “grow from seeds” with seeds being placed to identify stroke lesion areas and non-stroke areas. After seed growth, smoothing was applied, and the resulting volume was recorded. The same method was used on a separate copy of the scan to quantify ventricular volumes.

### 2.8 Tissue processing

All dogs were anesthetized using isoflurane and euthanized through perfusion with PBS and 4% PFA, then decapitated. After being removed, their brains were incubated in 4% PFA for 24 h at 4°C. The next day, using a shaker, the brains were washed with PBS three times for 5 min each. The brains were immersed in 10% sucrose for 24 h at 4°C to create a sucrose gradient. The brains were then immersed in 20% sucrose for 24 h at 4°C the next day. Finally, the brains were immersed in 30% sucrose for 24 h at 4°C the following day.

### 2.9 Tissue cryosectioning

The dogs' brains were embedded in OCT the next day, and 20 um thick brain cryo-sections were made using a Leica CM1850 cryostat.

### 2.10 Tissue staining

Sections were blocked with 5% donkey serum, permeabilized with PBST (0.3% Triton X-100), and fixed with 4% PFA. After that, slices were incubated with primary antibodies against GFAP (1:500, abcam, ab278054), NeuN (1:500, abcam, ab104224), Iba1 (1:50, Wako Chemicals, 019-19751), and NeuroD1 (1:250, abcam, ab205300) overnight at 4°C in 5% donkey serum blocking solution. Sections were washed with PBST (0.1% Triton X-100), let to sit at room temperature for an hour, and then centrifuged in 5% donkey serum blocking solution with secondary antibodies labeled with Alexa Fluor 488, 555, or 647 (1:1000, Abcam). Following rinsing, the slices were wet mounted and imaged using a Leica DMI6000 B microscope.

### 2.11 Co-labeling

To detect mRuby2 co-labeling, analysis of microscopy acquired images was performed using QuPath. Cell detection was performed on channel wavelength 555, to identify all mRuby2 positive cells. After identification, single measurement classifiers were used to classify if a cell was co-labeled for NeuN or GFAP. Classifiers were determined per image and changed depending on staining quality. After classification, the resulting values were recorded, and statistics were performed.

## 3 Results

### 3.1 Experimental design

**Figure 2:**
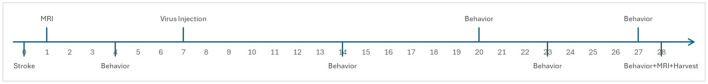
Timeline for astrocyte to neuron reprogramming canine stroke models experiment.

Established reports in the scientific literature have shown beneficial effects of the use of cellular reprogramming in stroke models (Chen et al., 2020). We decided to explore larger, more comprehensive animal stroke models with vasculature more analogous to humans (i.e., canine). For this experiment, 3 male beagle canine stroke models were used, 2 of which were given the treatment viruses “AAV9-GFAP(short)-Cre: 2.4E13 GC/mL and AAV9-CAG-DIO-NeuroD1-T2A-mRuby2: 1.0E13 GC/mL” meant to reprogram astrocytes into neurons and 1 of which was given the control viruses “AAV9-GFAP(short)-Cre: 2.4E13 GC/mL and AAV9-CAG-DIO-mRuby2: 2.5E13 GC/mL” meant to identify transduced cells. Stroke was induced at day zero, an MRI was acquired at day 1, the virus was injected at day 7, a second MRI was taken at day 28, and the animals were euthanized at day 29 (Figure 2). Periodically through that time behavioral data was also acquired.

### 3.2 Stroke lesion volume

**Figure 3:**
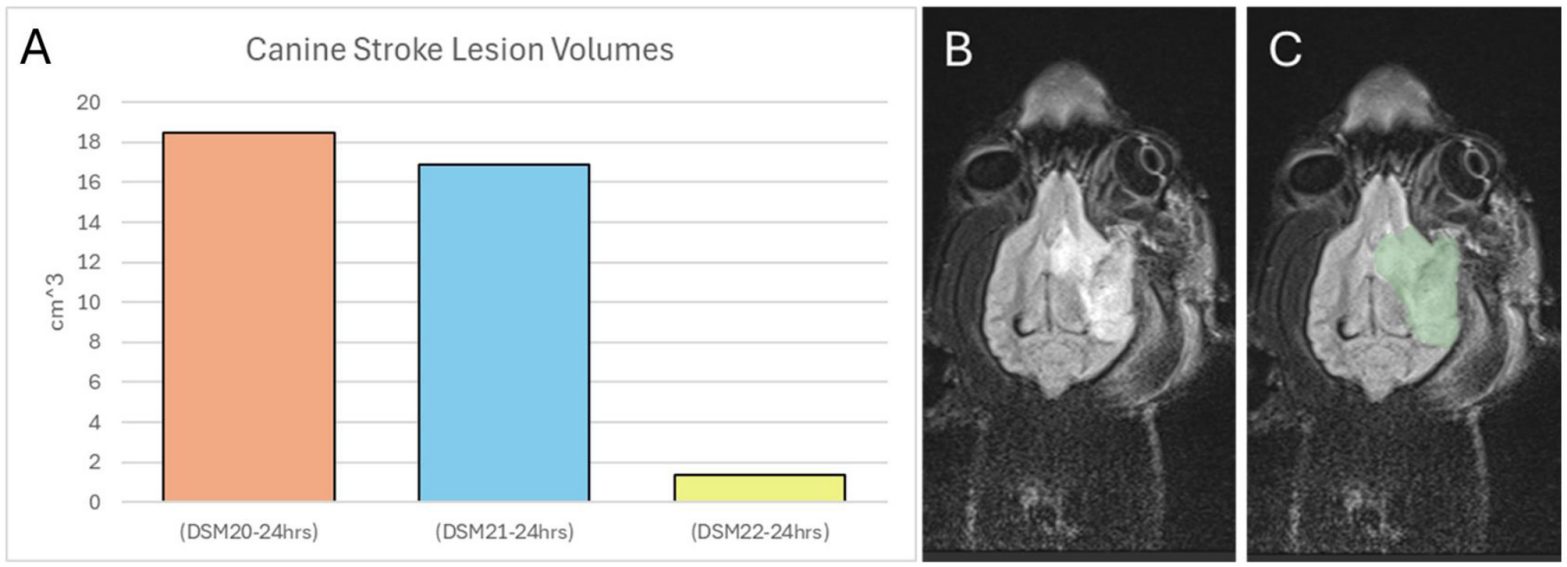
Canine stroke lesion volumes. **(A)** Volume in cm^3^ of each animal's stroke. **(B)** T2 MRI scan of canine brain 24 h post stroke. **(C)** Segmented stroke volume of T2 scan.

Stroke lesion volume was quantified using the T2 Flair MRI scan from day 1. The quantification was performed using segmentations in 3D Slicer. This quantification revealed that two canines had large strokes while one had a significantly smaller stroke (Figure 3). Due to this significant difference, animal DSM21 (one of the large stroke animals) was chosen as the control animal, while the other two were administered the treatment virus. Additionally, to account for the varying stroke lesion volumes, the data were quantified individually for each animal rather than averaged.

### 3.3 Hemisphere volumes

**Figure 4:**
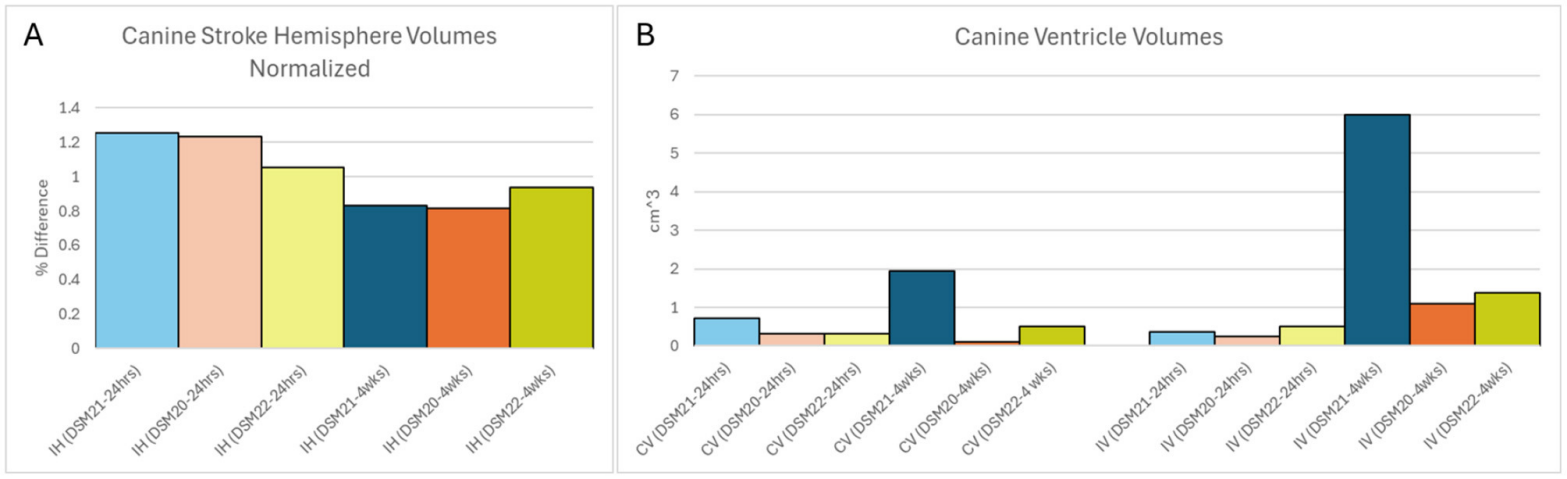
Anatomical changes over time. **(A)** Canine Stroke Hemisphere Volumes Normalized. The ipsilateral hemisphere was normalized against the corresponding contralateral hemisphere. IH, Ipsilateral Hemisphere; Blue, Control animal; Orange, Treated animal with large stroke; Yellow, Treated animal with small stroke; Lighter colors representing 24 h post stroke and darker colors representing 4 weeks post stroke. **(B)** Canine Ventricles Volume. IV, Ipsilateral Ventricle; CV, Contralateral Ventricle.

The left and right hemispheres of each canine were quantified using AX Recon MRI scans from day 1 and 28. Hemisphere quantification allows for the analysis of inflammation or tissue loss between the hemisphere on the ipsilateral side of the stroke and the contralateral side. The ipsilateral hemisphere from each animal and time point was normalized to the corresponding contralateral hemisphere to account for any quantification error (Figure 4A). Normalization was performed by dividing the total volume of the ipsilateral hemisphere (in cm^3^), by the total volume of the contralateral hemisphere (in cm^3^). This quantification revealed that the ipsilateral hemisphere of each animal was larger than the contralateral hemisphere at 1 day post-stroke. This correlates well with the existing literature (Anrather and Iadecola, 2016), as strokes induce inflammation and swelling in the affected brain region. Prior to treatment, similar levels of swelling were observed in the large stroke animals while much less swelling was seen in the small stroke animal. Specifically, large stroke animals DSM20 and DSM21 exhibited ipsilateral hemispheres that swelled to 123% and 125% the size of their corresponding contralateral hemisphere, respectively, while the small stroke animal DSM22 showed swelling of only 106%. On day 28, after sufficient time for brain recovery and treatment administration, the ipsilateral hemisphere in all animals was smaller than the corresponding contralateral hemisphere indicating atrophy or tissue loss within the ipsilateral hemisphere. Interestingly there was little difference between the large stroke-treated animal DSM20 and the large stroke-control animal DSM21 with their ipsilateral hemispheres being 81% and 83% the size of their corresponding contralateral hemispheres, respectively. Similarly, the small stroke-treated animal exhibited less difference in hemisphere sizes with its ipsilateral hemisphere being 93% the size of its corresponding contralateral hemisphere.

### 3.4 Ventricle sizes

The left and right ventricles of each canine were quantified using the AX Recon MRI scans from days 1 and 28 (Figure 4B). Ventricular enlargement is often associated with tissue atrophy, blockage, and/or changes in tissue properties such as decreased diffusivity (Sayed et al., 2020; Zahr et al., 2013). On day 1 post-stroke, ventricular sizes were similar between the contralateral and ipsilateral sides across all animals. However, by day 28 the ventricle on the ipsilateral side of all animals was noticeably larger than its corresponding contralateral side, indicating some degree of tissue atrophy in all animals. Interestingly, the large stroke control animal DSM21 exhibited a substantial increase in both contralateral and ipsilateral ventricles of 2.7 × and 16.2 × respectively, from day 1 to day 28. However, as the hemisphere changes do not reflect this difference in the form of greater tissue loss, it can be assumed that this increase in ventricle size in the control animal is not correlated with greater tissue atrophy. This notable increase in ventricular size is more likely due to a potential ventricular blockage or detrimental changes to its tissue properties. These observations suggest that either this particular stroke resulted in an unintended blockage, or the treatment had a beneficial effect in preventing detrimental changes to tissue properties.

### 3.5 Transduced cell phenotypes

**Figure 5:**
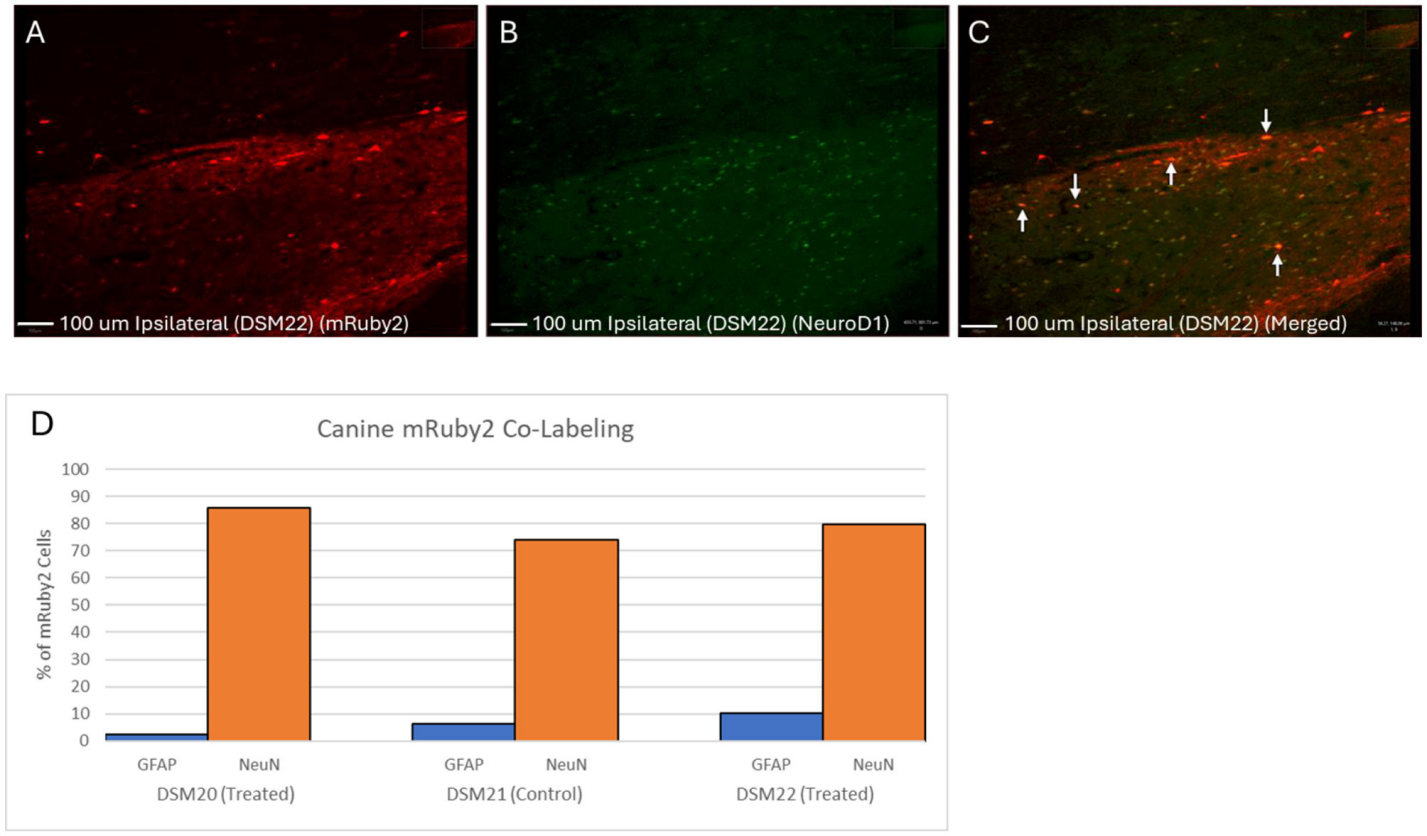
NeuroD1, NeuN, and GFAP co-labeling with mRuby2. **(A–C)** Canine NeuroD1 & mRuby2 Co-labeling in treated animal DSM22. **(A)** mRuby2 alone Red, mRuby2. **(B)** NeuroD1 alone Green, NeuroD1. **(C)** Merged. QuPath quantification revealed 70.95% of mRuby2 positive cells were positive for NeuroD1. **(D)** Canine mRuby2 Co-Labeling. Percentage of mRuby2 cells co-labeled with either GFAP or NeuN. Green, NeuN; Purple, GFAP.

To determine which cells were transduced and the rate of cellular reprogramming, tissue was cryosectioned and stained for GFAP and NeuN markers using IHC to identify astrocytes and neurons respectively (Figure 5D). To prevent image bleed through, slices were stained with either GFAP or NeuN, but not both. If reprogramming occurred, we would expect to see a majority of the mRuby2 co-labeled cells in the treated animals to be NeuN positive. Within treated animal DSM22, approximately ~80% of mRuby2 cells were co-labeled with NeuN. Within the treated animal DSM20, approximately ~85% of mRuby2 cells were co-labeled with NeuN. Interestingly, in control animal DSM21, despite the presence of the GFAP promoter and lack of the NeuroD1 sequence in the control virus, this animal also exhibited a high level of mRuby2 co-labeling in NeuN positive cells, at around 74%. This indicates that transduction may have targeted endogenous neurons rather than astrocytes or leakage may have occurred from the transduced astrocytes. As these experiments were conducted with high dose viral injections, these results align with previous research suggesting that injections of astrocyte-targeting viruses with higher doses often lead to more co-labeling with NeuN positive cells (Xu et al., 2022). There is however a noticeable increase in NeuN positive mRuby2 cells in the two treated animals with 80 and 85% vs. the control animal with 74%. Since NeuN is a neuronal marker, this increase suggests that some astrocytes were successfully reprogrammed into neurons. Although this suggestion remains inconclusive given the exploratory nature of the study, it represents a significant step forward that can serve as a foundation for future research. Further analysis of NeuroD1/mRuby2 co-labeling confirmed the absence of NeuroD1 in the control animal. Nevertheless, more than 70% of mRuby2 positive cells were NeuroD1 positive in treated animals (Figures 5A–C).

### 3.6 Astrocyte and microglial activation

**Figure 6:**
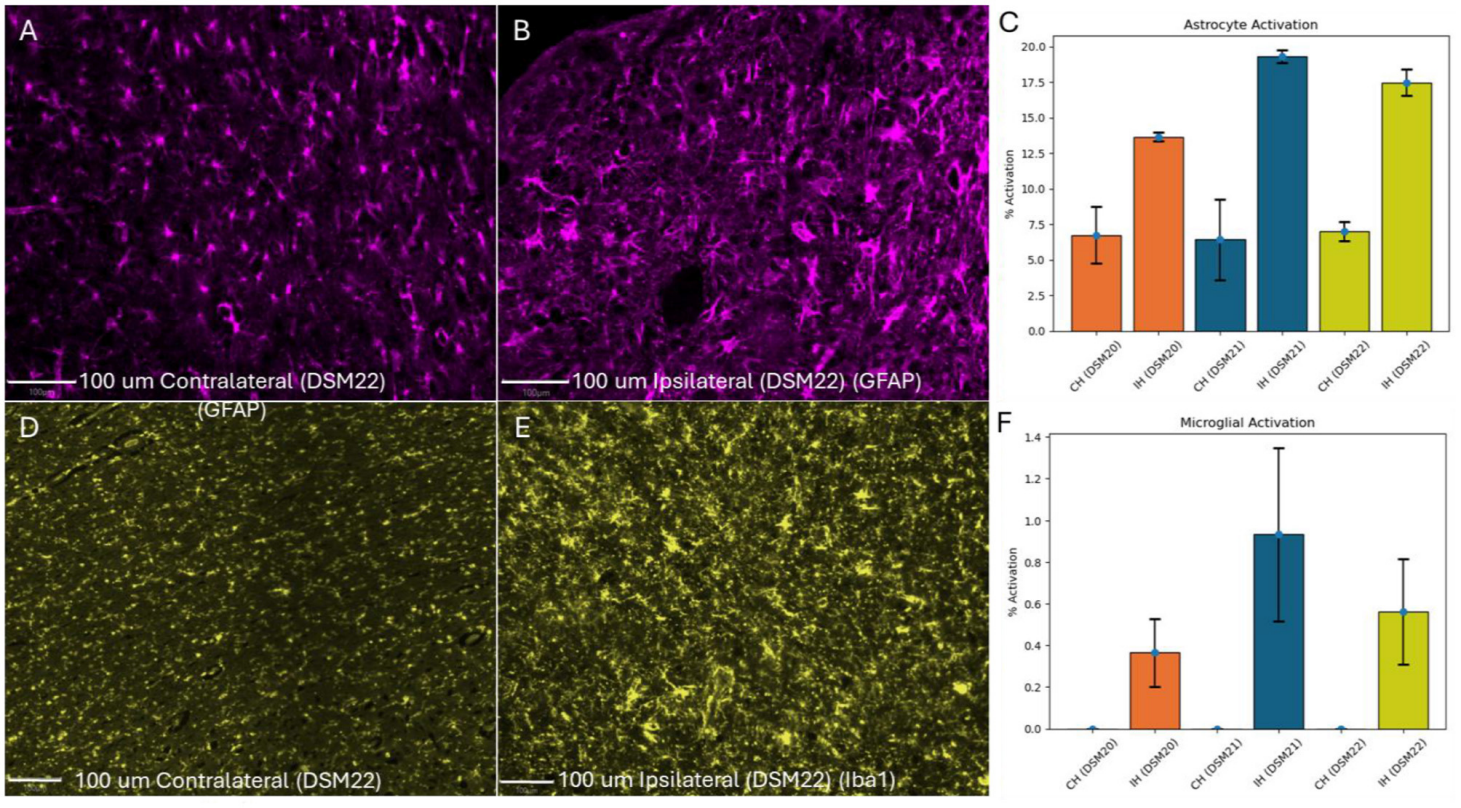
Astrocyte & microglial activation. **(A)** Contralateral Hemisphere of treated canine DSM22 with GFAP Staining (minimal enlarged astrocytes present). **(B)** Ipsilateral Hemisphere with GFAP Staining (several enlarged astrocytes present). **(C)** Number of activated astrocytes divided by total astrocytes. **(D)** Contralateral Hemisphere of treated canine DSM22 with Iba1 Staining (No activated microglia present). **(E)** Ipsilateral Hemisphere with Iba1 Staining (several activated microglia present). **(F)** Number of activated microglia divided by total microglia. CH, Contralateral Hemisphere; IH, Ipsilateral Hemisphere; Blue, Control animal; Orange, Treated animal with large stroke; Yellow, Treated animal with small stroke.

To assess the immune response in all animals, sections near the stroke site stained for GFAP were quantified for astrocyte activation and microglial activation (Figures 6A–F). Images were acquired near the stroke lesion and the corresponding contralateral side. Five regions were quantified on both the ipsilateral and contralateral sides for each animal. Activated astrocytes typically exhibit enlarged somas and additional processes (Pekny et al., 2014). To distinguish between inactive and active astrocytes, we measured the average soma size of astrocytes in the contralateral (unaffected) hemisphere and calculated its standard deviation. We then defined a threshold for activation as two standard deviations above this average (Threshold = Average + 2 × Standard Deviation). Astrocytes with soma sizes exceeding this threshold were considered statistically unlikely to belong to the inactive group, indicating they were active. We then estimated the percentage of active vs. inactive astrocytes and found that the control animal had the highest rate of astrocyte activation in the ipsilateral hemisphere (19%) (Figure 6C). Similarly, activated microglia tend to be enlarged, and we used the detection data to estimate a threshold size value to differentiate them. The microglial activation followed the same trend as the astrocyte activation with greatest activation in the ipsilateral side of the control animal (Figure 6F).

### 3.7 Behavioral analysis

**Figure 7:**
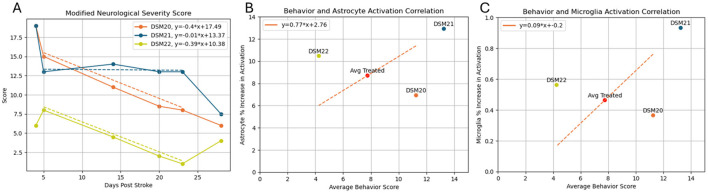
**(A)** Modified neurological severity score vs. days post stroke. Trendlines generated from day 5–23 range. **(B)** Average behavior score vs. astrocyte activation. **(C)** Average behavior score vs. microglia activation. Blue, Control animal; Orange, Treated animal with large stroke; Yellow, Treated animal with small stroke.

To assess functional recovery, the behavior of each animal was evaluated using a modified neurological severity score (Figure 7A). All animals showed neurological impairments after stroke and had some level of recovery in the following days. As expected, the smaller stroke animal DSM22, had fewer impairments compared to the animals with larger strokes. Promisingly, the large stroke-treated animal DSM20 showed faster and greater overall recovery than the large stroke control animal DSM21. Between days 5 and 23, it can be noticed that the treated animals had a nearly identical linear recovery, with trendline slopes of −0.401 and −0.393, while the control animal remained almost stagnant in recovery with a trendline slope of −0.016. This coincides with the timing of viral injection which occurred on day 7 and suggests the treatment's presence had some beneficial neurological effects and provide a basis for future research. When comparing the average behavior score to the microglia and astrocyte activation, we get two trend lines supporting that greater inflammatory responses correlates to worse neurological severity scores (Figures 7B, C).

## 4 Discussion

### 4.1 Stroke lesion volume

The stroke lesion volumes in canines were assessed using T2 FLAIR MRI scans and segmentations in 3D slicer, revealing significant differences among the subjects. This method aligns with established practices in stroke research, where precise quantification of lesion volumes is crucial for evaluating the extent of ischemia damage and its impact on treatment outcomes. Prior studies have demonstrated the reliability of MRI-based volumetric analysis in stroke models, which is critical for comparing control and treatment groups (Heiss and Zaro Weber, 2016). Given the substantial variation in lesion sizes, individualized data quantification was necessary rather than averaging.

### 4.2 Hemisphere volumes

The enlargement of the ipsilateral hemisphere relative to the contralateral side at 1 day post-stroke is consistent with the well documented inflammatory response following ischemic injury. This swelling, as noted, typically reduces overtime due to tissue atrophy and repair processes. The literature supports that stroke-induced inflammation and subsequent atrophy are common, contributing to volume changes in the affected hemispheres (Anrather and Iadecola, 2016).

### 4.3 Ventricle sizes

The quantification of ventricle sizes revealed the enlargement of the ipsilateral side from day 1 to 28 post-stroke, this is indicative of tissue atrophy and potential ventricular blockage. This finding correlates with studies that showed ventricular enlargement as a common consequence of brain atrophy and changes in brain tissue properties following a stroke (Sayed et al., 2020; Zahr et al., 2013). The significant increase in ventricle size in the control animal (DSM21) suggests a potential ventricular blockage or other detrimental changes not observed in the treated animals, highlighting the potential benefits of the treatment in preventing such adverse effects.

### 4.4 mRuby2 expression and co-labeling

The high rate of mRuby2 co-labeling with NeuN in both treated and control animals suggests a degree of neuronal targeting by the viral vector, despite the intention to target astrocytes. This outcome may result from the use of high-dose virus injections, which can increase off-target effects. Despite the use of high dose, an increase in NeuN positive transduced cells was identified along with the expression of NeuroD1, suggesting that reprogramming occurred in the treated animals. However, this evidence alone is insufficient to confirm true reprogramming. Future experiments should incorporate lineage tracing to verify the astrocyte origin of newly generated neurons. Additionally, to address potential toxicity, studies should be conducted to track cell death across varying viral doses.

### 4.5 Astrocyte and microglial activation

The immune response across all three animals was similar with low to no astrocyte and microglial activation in the contralateral sides and higher activation in the ipsilateral side near the stroke lesion. Interestingly, the treated animals had lower levels of activation in the ipsilateral side than the control animal. This is suggestive of the treatment being able to lower astrocyte and microglia activation, ultimately reducing the neuroinflammation in treated animals. It is possible that with the addition of NeuroD1, some of the astrocytes that would have been activated, were reprogrammed into neurons instead. Activated astrocytes produce proinflammatory cytokines such as interleukin which activate microglia (Van Wagoner et al., 1999; Xiong et al., 2022). As a consequence of less activated astrocytes, less pro-inflammatory cytokines would be present, likely leading to the reduction in microglia activation that we saw in treated animals. This will be explored in future studies through staining for cytokines such as interleukin-6 (IL-6) and tumor necrosis factor-alpha (TNF-α).

### 4.6 Behavioral

The modified neurological severity score used to assess functional recovery indicated that the treated large stroke animal (DSM20) exhibited faster rate and greater recovery compared to the control (DMS21). As expected, the treated small stroke animal had the best behavior scores with fastest and greatest levels of recovery. Although the sample size limits statistical significance, these findings provide a promising basis for further research on the efficacy of the treatment. This observation aligns with studies demonstrating that interventions aimed at reducing inflammation by blunting the activation of astrocytes and microglia, and promoting neurogenesis can improve functional outcomes after stroke, as Lee et al. have shown that early intervention and targeted therapies can significantly enhance recovery in post-stroke models.

### 4.7 Limitations

While this study provided promising exploratory results, several limitations must be acknowledged. The small sample size, comprising one large stroke control animal, one large stroke treatment animal, and one small stroke treatment animal, precludes statistically significant conclusions. The inclusion of the small stroke animal also complicates interpretation, as the reduced severity of injury makes direct comparison with the large stroke animals difficult and introduces a potential confounding variable. Moreover, the study lacked a true negative control group; even the control animal received a virus injection, which may have influenced outcomes independently of the treatment. The reliance on microglial and astrocyte activation to describe levels of neuroinflammation, without cytokine analysis, further limits the precision with which the immune response can be characterized. Lastly, the absence of retrograde labeling prevents confirmation that the neurons observed post-treatment originated from astrocytes, leaving the proposed mechanism of astrocyte-to-neuron conversion unverified.

### 4.8 Future work

Future studies should include a larger sample size to enable statistically significant findings. In addition, incorporating a true negative control group—receiving no injection—will help isolate the specific effects of the treatment from any procedural influences. Further investigation should also involve cytokine profiling to more precisely characterize the immune response. Measuring key inflammatory markers such as interleukin-6 (IL-6) and tumor necrosis factor-alpha (TNF-α) would provide greater insight into the nature and magnitude of the inflammatory processes involved. Finally, to confirm that the observed neurons are indeed derived from astrocytes, retrograde labeling should be employed. For example, the use of Aldh1l1-Cre transgenic animals—where Cre recombinase is expressed in Aldh1l1-positive astrocytes prior to viral injection—would allow lineage tracing (Srinivasan et al., 2016). The presence of Cre in resulting neurons would confirm their endogenous astrocytic origin.

## 5 Conclusion

This study evaluated anatomical recovery of stroke through MRI and 3D segmentation. We found that ipsilateral hemisphere enlargement in all animal models was consistent with the inflammatory response typically seen in ischemic injury. Ventricle enlargement was consistent with literature describing post ischemic injury effects, with the control animal showing greater enlargement than treated. This suggested beneficial effects of the treatment on tissue atrophy. Co-labeling studies revealed mRuby2 to be highly co-localized with NeuN in both control and treated, indicating that unintended neuronal targeting took place. This is likely due to the high dose virus injections and resulting in leakage. Although reprogramming likely still occurred in the properly targeted astrocytes that survived, we cannot be confident in attributing the beneficial effects solely to them. The inflammatory response appeared to be upregulated in the control animal while treated animals had less neuroinflammatory activation. This indicates that the treatment is able to mitigate the astrocyte and microglia activation which could explain the lessened ventricle enlargement in treated animals. The likely mechanism behind this lessened inflammatory response is the conversion of astrocytes into neurons that otherwise would have been activated, leading to less pro-inflammatory cytokine production and less astrocyte and microglial activation. Behaviorally, the treated stroke animals recovered faster and to a greater extent compared to the control. This may also be explained by decreasing microglia and astrocyte activation where decreases in neuroinflammation are associated with improved cognitive performance. Collectively, these findings provide evidence that cell reprogramming with NeuroD1 can modulate the neuroinflammatory response associated with stroke and impact functional recovery in a canine model.

## Data Availability

The original contributions presented in the study are included in the article/supplementary material, further inquiries can be directed to the corresponding author.
